# Adaptation and validation of the HIV Knowledge Questionnaire-18 for the general population of Indonesia

**DOI:** 10.1186/s12955-022-01963-5

**Published:** 2022-04-02

**Authors:** Bustanul Arifin, M. Rifqi Rokhman, Zulkarnain Zulkarnain, Dyah Aryani Perwitasari, Marianti Manggau, Saidah Rauf, Rasuane Noor, Retna Siwi Padmawati, Maarten J. Postma, Muhammad Nasrum Massi, Jurjen van der Schans

**Affiliations:** 1 Faculty of Pharmacy, Universitas Hasanuddin, Makassar, Indonesia; 2 Unit of Global Health, Department of Health Sciences, University of Groningen, University Medical Centre Groningen (UMCG), Ant. Deusinglaan 1, 9713 AV Groningen, The Netherlands; 3 Unit of PharmacoTherapy, Epidemiology and Economics (PTE2), Department of Pharmacy, University of Groningen, Groningen, The Netherlands; 4 Institute of Science in Healthy Ageing and healthcaRE (SHARE), University Medical Center Groningen, University of Groningen, Groningen, The Netherlands; 5 Faculty of Pharmacy, Universitas Gadjah Mada, Yogyakarta, Indonesia; 6 Faculty of Medicine, Universitas Syiah Kuala, Banda Aceh, Indonesia; 7 Thyroid Center, Zainoel Abidin Hospital, Banda Aceh, Indonesia; 8 Faculty of Pharmacy, Universitas Ahmad Dahlan, Yogyakarta, Indonesia; 9 Masohi Nursing Study Program, Politeknik Kesehatan Kemenkes Maluku, Ambon, Indonesia; 10 Universitas Muhammadiyah Metro, Lampung, Indonesia; 11 Department of Health Behaviour, Environment, and Social Medicine, and Centre of Health Behaviour and Promotion, Faculty of Medicine, Public Health and Nursing, Universitas Gadjah Mada, Yogyakarta, Indonesia; 12 Department of Economics, Econometrics and Finance, Faculty of Economics and Business, University of Groningen, Groningen, The Netherlands; 13 Department of Pharmacology and Therapy, Faculty of Medicine, Universitas Airlangga, Surabaya, Indonesia; 14 Center of Excellence in Higher Education for Pharmaceutical Care Innovation, Universitas Padjadjaran, Bandung, Indonesia; 15 Department of Microbiology, Faculty of Medicine, Universitas Hasanuddin, Makassar, Indonesia

**Keywords:** Infectious disease, Education, Psychometric properties, General population, Instrumental study

## Abstract

**Background:**

Despite a global decline in new HIV/AIDS cases in low-middle countries, cases are increasing in Indonesia. Low knowledge about the disease among the general population is one of the major factors responsible for this trend. Indonesia does not have a validated instrument to assess HIV/AIDS knowledge. The HIV Knowledge Questionnaire-18 (HIV-KQ-18) has been translated into several languages and is one of the most extensively used instruments for assessing HIV/AIDS knowledge. This paper describes the process of adapting and validating the HIV-KQ-18, an instrument to assess the level of HIV/AIDS knowledge in the general population of Indonesia.

**Methods:**

In the adaptation phase, feedback for the initial Bahasa Indonesia version was gathered from two HIV activists, an obstetrician, two general practitioners, and 60 pilot participants. At the validation stage, we distributed the instrument link via Google Form to 6 major regions in Indonesia. Validity was measured using known-group validity and construct validity. The construct validity was assessed using an exploratory factor analysis (EFA) with a polychoric correlation matrix. Cronbach’s alpha was used to analyze the internal consistency.

**Results:**

Based on the findings in the adaptation phase, additional descriptions (namely synonyms or examples) were added to 6 items to make them more understandable. In the validation phase, 1,249 participants were recruited. The a priori hypothesis in known-group validity was supported. We also found three items that did not meet the construct validity. Based on the acceleration factor approach to interpret the scree tree in the factor analysis, using only two factors was preferable. Cronbach's alpha values were 0.75 and 0.71 representing good internal reliability.

**Conclusion:**

The HIV-KQ-18 Bahasa Indonesia is considered a valid and reliable instrument to assess the level of HIV/AIDS knowledge in Indonesia.

## Introduction

The Human Immunodeficiency Virus (HIV) and Acquired Immunodeficiency Syndrome (AIDS) remain a major global health crisis and the intense efforts in international and local initiatives are continuing to mobilize resources to fight the HIV/AIDS pandemic [1]. The Joint United Nations Programme on HIV/AIDS (UNAIDS) estimates that in 2019 there were 38.0 million people infected with HIV worldwide, and 36.2 million of them were adults (more than 15 years). Additionally, it is estimated that there are currently around 7.1 million people who are not conscious that they have been infected with HIV [2]. The millennium development goals (MDGs) initiated a concerted global effort to resolve the increasing spread of HIV/AIDS [3]. There is an urgent need to develop more proactive programs with prevention education to stem the rising incidence of HIV, and the global community needs to re-evaluate the MDGs’ progress across all world regions.

The majority of HIV patients live in low to middle-income countries such as Indonesia [4]. In Indonesia, at the end of 2018, the number of people with HIV/AIDS was 640,000 cases of which 46,000 were new cases [5]. The top five provinces with the highest number of HIV/AIDS patients are DKI Jakarta, East Java, West Java, Papua, and Central Java [6]. The number of deaths due to HIV/AIDS increased by around 60% from 24,000 to 38,000 between 2010 and 2018 [5].

The results of a study (RISet KESehatan DASar/RISKESDAS) conducted by the Ministry of Health of the Republic of Indonesia reported that one of the main factors causing the increase in the number of HIV cases in Indonesia is the low level of knowledge of HIV/AIDS [7]. Behavioral change provides the ultimate and cheapest protection against HIV infection since no cure nor vaccine are available for HIV, and people with less HIV knowledge are more likely to engage in more risky sexual behaviors [8]. The Ministry of Health mapped the level of knowledge of Indonesians based on the 34 provinces in Indonesia in 2018. Based on this mapping, the level of knowledge of HIV/AIDS in Indonesia was still very low: 67% scored below standard (< 7 of 21 points) and only 1% scored high-level knowledge (> 16 of 21 points) [7]. However, this report did not specify the type of instrument used to measure the level of knowledge.

HIV-KQ-18 is available in many languages and already implemented in other settings, such as Spanish, Greek and Indonesia [9]. Two studies in Indonesia have used the HIV-KQ-18 instrument to assess the level of knowledge in Indonesia. These two studies focused on specific communities, namely on 120 women living with HIV/AIDS in Lampung [10] and 396 nurses who worked in four hospitals in Jakarta [11]. However, neither of these studies performed or reported a psychometric test of the HIV-KQ-18, except for the study in Lampung reporting only on the reliability test with Cronbach’s alpha [10]. No studies exist to fully adapt and validate this instrument for the Indonesian general adult population.

The HIV-KQ-18 instrument has been proven to be a valid (good internal consistency, Cronbach’s alpha at 0.75—0.89), stable, sensitive, and appropriate instrument for all people including low-literacy populations [12]. An instrument to assess the HIV/AIDS knowledge level is important to indicate in which specific aspects the public needs to improve, to develop content for effective campaigns, and to assess the knowledge trend from time to time as an indicator to measure the success of an HIV campaign. Therefore, the study aimed to conduct an adaptation, validation, and psychometric test of the HIV-KQ-18 instrument on a general adult population in Indonesia.

## Methods

### Research design

The study used a cross-sectional design. Data were collected from September 2020 to January 2021. The study was approved by the Research Ethics Committee of Universitas Ahmad Dahlan, Yogyakarta, with ethical approval number 012007028 on 22 September 2020 and was divided into two main phases, adaptation and validation.

### Instrument

The instrument consisted of sociodemographic characteristics and the HIV-KQ-18 instrument. HIV-KQ-18 is the short version of HIV-KQ-45 [9, 13]. Permission to translate the HIV-KQ-18 instrument was obtained from Prof. Michael P. Carey, PhD (Director, Centers for Behavioral and Preventive Medicine, The Miriam Hospital) on February 11, 2020. The HIV-KQ-18 instrument is more focused on how to prevent infection and transmission of HIV/AIDS. This instrument consists of 18 items, and each item has 3 options, namely “true”, “false” or “don’t know”. Five items (no 1, 4, 11, 14, 17) are true statements, while the other 13 items are false. The correct response is scored 1, while 0 is used for wrong or “don’t know” responses.

We collected sociodemographic data included gender, age, occupation, education level, marital status and monthly expenses. We asked for monthly expenditure data instead of monthly income since participants indicated that they were more comfortable reporting expenses than income. Participants were classified concerning having an educational background in health sciences (medicine, pharmacy, nursing, midwifery, and public health) and whether they had attended a workshop about a HIV/AIDS education. To maintain confidentiality, participants were given the right to only write their name initials and age. Only researchers had the right to access the dataset.

### Cross-cultural adaptation and validation

Indonesian researchers conducted a literature review and agreed to continue the study of the HIV-KQ-18 instrument's validation with several caveats, including: (i) Indonesia does not yet have a validated instrument that can be used to assess HIV/AIDS knowledge; and (ii) there are some very interesting questions that need to be explored further, such as condom knowledge and the method of transmitting HIV/AIDS. Based on our findings and experiences, these two themes are still relatively rarely discussed in public. The original HIV-KQ-18 instrument was translated to Bahasa Indonesia using a forward–backward translation. As a result, the Indonesia research team is continuing to adaptation processes.

The research team consists of scientists and professionals in the fields of HIV, health, and psychometrics study. There are nine Indonesian authors in the team and their origins also represent three regions of Indonesian (West, Central and East Indonesia). Five authors are from Western Indonesia (MRR, Z, DAP, RN, and RSP), three authors from the Central Region (BA, MM, and MNM), and one author from the Eastern Region (SR). Because of the various dialects in each region, this representation can reduce the chance of bias or ambiguity. In addition, DAP and BA are Indonesian researchers who have conducted similar studies several times. Furthermore, all Indonesian authors are lecturers, and lecturers in Indonesia are required to 'do community service.' The majority of them have taken part in HIV/AIDS education programs for the community, particularly for middle and high school teachers and students. Several of the authors have conducted at least one similar study (some are in the process of submitting manuscripts and some are in the process of collecting data).

**Fig. 1:**
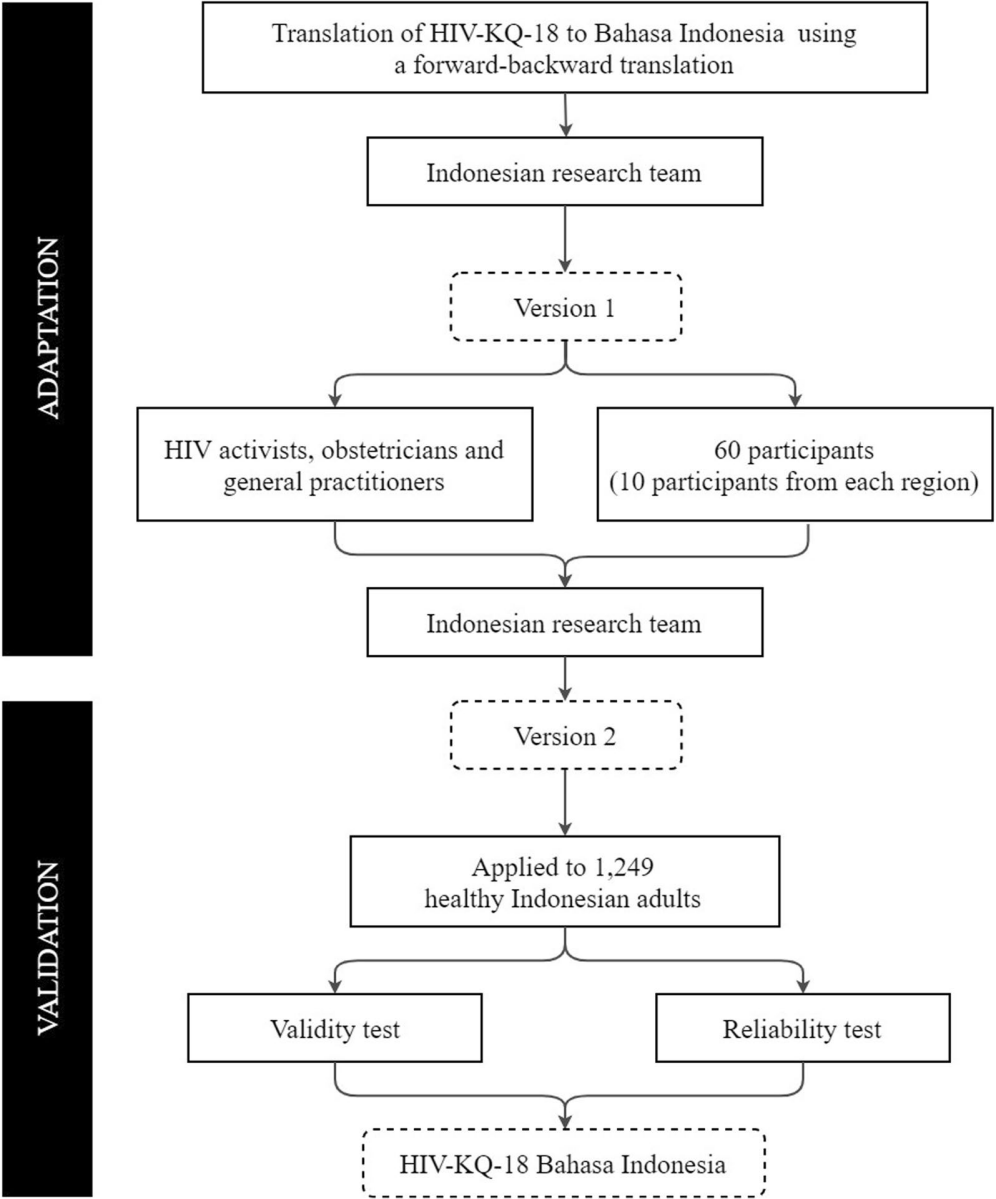
Adaptation and validation of HIV-KQ-18 for Indonesian population

Some psychometric study components, such as content validity, face validity, and construct validity [14–16], were investigated in this study. As illustrated in Fig. 1, an expert panel analyzed and consolidated the Indonesian translation of the original version in order to obtain consensus on inconsistencies and produce a version 1 for field testing to 60 participants. At the end of the instrument, we added a question “Out of the 18 items, which statement was the most difficult or took a long time to answer?”. This question was used to identify additional obstacles by participants to understand the HIV-KQ-18 Bahasa Indonesia instrument.

### Participants

Participants were Indonesians at least 17 years old, who consented to participate in the study. Participants involved in the study were sampled from six of Indonesia's main regions, namely: Sumatra, Java, Sulawesi, Kalimantan, 'Bali and Nusa Tenggara', and 'Maluku and Papua'. It is important to highlight that the 'research site' in this study refers to the participant's domicile location (district and province) as submitted on the online link that we distributed.

### Sample size

The sample size calculation was based on a study suggesting at least 100 participants should be the minimum limit for psychometric study [17]. Two previous studies stated that the minimum number of participants should be 200 if the number of items in the instrument is not more than 40 [18, 19]. Another study recommends that the minimum number of participants involved in a psychometric study should be the number of items in the instrument to be validated multiplied by ten [20]. Accordingly, the minimum number of participants for each region included in the study is 180 (18 items × 10). *Ergo*, the minimum total of participants in this study was set at 1080 (180 × 6) participants.

### Data collection

The initial Indonesian version of HIV-KQ-18, after forward and backward translations, was evaluated by the Indonesian research team. After obtaining ethical clearance, in the adaptation phase we requested feedback for the initial version of the HIV-KQ-18 from HIV activists, an obstetrician, general practitioners and 60 pilot participants (10 participants from each region). Feedback was reviewed by the Indonesian research team to develop the final version of HIV-KQ-18 Bahasa Indonesia.

In the validation phase, we distributed the online link of the final version of HIV-KQ-18 Bahasa Indonesia through several social media from 29 September 2020 to 6 April 2021, namely: WhatsApp, Facebook inbox, email, Instagram, and Twitter. If participants joined through various media, we removed the duplicate participants based on the initials and date of birth. Figure 1 provides an overview of the study procedure.

### Data analysis

During the adaptation phase, we discussed all of the participants’ suggestions. Furthermore, the Indonesian core research team compiled and analyzed the pilot data for the best choice of words and word order in one single sentence (item) through consensus. Notably, whenever differences emerged in this phase, these issues were resolved by consensus.

Participants’ characteristics in the adaptation phase were analysed descriptively. Item analysis was conducted by calculating the percentage of the correct answers of each item and corrected item-total correlation. Items with the percentage of correct answers being between 30 and 80% were considered appropriate [21], because it avoids floor and ceiling effects and allows for additional scores to capture knowledge gains after an education program. Items with a corrected item-total correlation lower than 0.3 were considered to indicate that the items were candidates for deletion [22]; however, an item with a corrected item-total correlation higher than 0.25 was still considered acceptable.

Validity and reliability tests were used to analyze the psychometric properties of HIV-KQ-18 Bahasa Indonesia. Cronbach’s alpha was used to analyze the internal consistency, and a Cronbach’s alpha of higher than 0.7 is considered as a reliable instrument [22].

Validity was measured using known-group validity and construct validity. Known-group validity was determined by comparing the HIV knowledge between participants based on four variables, namely education level, educational background, experience in attending a workshop about HIV and monthly expenses. Previous studies conducted in the Asian general population have demonstrated that different subgroups based on education levels [23–29], HIV education [28] and socioeconomic status [23, 24, 27–29] have different HIV knowledge levels. Therefore, the a priori hypothesis was made that participants with higher education level, educational background in health sciences, experience in attending a workshop about HIV and higher monthly expense had significant higher HIV knowledge than participants with lower education level, without educational background in health sciences, never experience in attending a workshop about HIV and lower monthly expense. The difference of HIV knowledge levels was assessed using an independent t-test for a variable with two subgroups, while ANOVA test for comparing HIV knowledge for a variable with more than two subgroups. If the result from ANOVA was significant, a post hoc analysis was carried using a Bonferroni test.

HIV-KQ-18 has dichotomous options; therefore, an exploratory factor analysis (EFA) using a polychoric correlation matrix was conducted to assess the construct validity [30, 31]. Muñoz-Quezada et al. (2021) used polychoric correlation to conduct factor analysis when validating an instrument with dichotomous options to measure the perception and knowledge about exposure to pesticides [32]. To eliminate the subjectivity in interpreting the scree plot, the number of factors that could be retained was determined based on eigenvalues, parallel analysis, optimal coordinates, and acceleration factor. If these parameters had different recommendations regarding the number of factors to be retained, other considerations were applied in which a factor should have at least 3 items [20] and the possibility of underlying factors to be interpreted. Goodness of fit was assessed using the root mean square of residuals (RMSR) and the Root Mean Square Error of Approximation (RMSEA). A factor loading of 0.4 or higher is required to indicate a good relationship between each item and underlying factor [20].

All statistical analyses were performed using the Statistical Package for Social Sciences (SPSS) version 26 (IBM Corp, Armonk, New York, USA), except for the EFA that was analyzed in R version 4.1.0 and RStudio Version 1.4.1717 using packages of polycor, nFactors and psych. The level of statistical significance was set at *p* < 0.05. Atlas.ti (Scientific Software Development GmbH, Berlin, Germany) was used to analyze the feedback from the participants regarding the most difficult item in responding to the HIV-KQ-18 Bahasa Indonesia.

## Results

### Participant characteristics

In total, 1,249 participants were recruited from six regions in Indonesia during the validation phase. All participants were those in the productive age range, predominantly females, with the majority of participants' monthly expenses being < 2 million rupiah (USD 137). The details of the sociodemographic characteristics of the participants are presented in Table 1.

**Table 1:** Participants’ characteristics

Variables	n	%
Total participants	1,249	100
Age		
18–25	728	58.3
25–35	336	26.9
35–45	185	14.8
Gender		
Female	715	57.2
Male	534	42.8
Education level		
Up to senior high school	315	25.2
Bachelor	804	64.4
Postgraduate	130	10.4
Marital status (n = 1201)		
No	812	67.6
Yes	389	32.4
Monthly expense (in Rupiah) (n = 1160)		
< 2 million	718	61.9
2–3 million	168	14.5
3–4 million	100	8.6
4–5 million	72	6.2
> 5 million	102	8.8
Having educational background in health sciences (n = 1242)		
No	578	46.5
Yes	664	53.5
Have attended workshop(s) about HIV (n = 1246)		
No	811	65.1
Yes	435	34.9
Location		
Sumatra	204	16.3
Java	217	17.4
Kalimantan	200	16.0
Bali and Nusa Tenggara	209	16.7
Sulawesi	209	16.7
Maluku and Papua	210	16.8

### Adaptation

After collecting feedback from participants during the adaptation phase, Table 2 indicates some improvements in the sentence structures or word selections. In total, there were 6 (six) items to which adjustments were made. The language patterns were adjusted by changing word selection, adding synonyms, or giving an example so that the item's context could be correctly interpreted by a broader range of participants. For example, some participants were more familiar with the term “climax”, while others did not understand that word and were more familiar with the term “orgasm”; therefore, we added the Indonesian term for orgasm.

**Table 2:** Revision of HIV-KQ-18 items according to adaptation phase

Item	Original version	Results of forward and backward translation in Indonesian (original article)	The final Indonesian version (along with a note of the changes we have made)
3	Pulling out the penis before a man climaxes/cums keeps a woman from getting HIV during sex	*Menarik penis sebelum seorang pria mencapai klimaks / mengeluarkan sperma mencegah wanita terkena HIV selama berhubungan seks*	*Menarik penis sebelum seorang pria mencapai klimaks/orgasme (sebelum mengeluarkan sperma); akan dapat mencegah seorang wanita terkena HIV selama berhubungan seks* (We added the word orgasm and explained the meaning of the word cum as the process by which the sperm comes out.)
4	A woman can get HIV if she has anal sex with a man	*Wanita dapat terkena HIV apabila dia berhubungan seks melalui anus dengan pria*	*Seorang wanita dapat tertular HIV jika dia melakukan hubungan seks anal (melalui dubur/anus) dengan seorang pria* (We added the words anal sex and dubur/rectum)
6	A pregnant woman with HIV can give the virus to her unborn baby	*Seorang wanita hamil dengan HIV dapat menularkannya kepada janinnya*	*Seorang wanita hamil penderita HIV, dapat menularkan penyakitnya kepada janin yang sedang dikandungnya. Hal ini berdampak pada bayi yang lahir akan menderita HIV seumur hidup* (We clarify the Indonesian version of the sentence by adding a few words. In addition, we added one sentence to clarify the statement in the previous sentence, namely “This has the impact that the baby will suffer from HIV for life”)
7	People who have been infected with HIV quickly show serious signs of being infected	*Orang yang telah terinfeksi HIV dengan cepat menunjukkan tanda –tanda terinfeksi yang serius*	*Orang yang telah terinfeksi HIV dengan cepat menunjukkan tanda-tanda serius sudah terinfeksi. Tanda-tanda serius ini, akan muncul maksimal 5 (lima) hari setelah terinfeksi* (We clarified the duration of the word quickly as 5 days or less.)
12	A natural skin condom works better against HIV than does a latex condom	*Kondom berbahan kulit alami bekerja lebih baik dalam melawan HIV daripada kondom berbahan karet*	*Kondom berbahan kulit alami (yang terbuat dari kulit domba atau lambskin) berfungsi lebih baik dalam melawan HIV dibandingkan dengan kondom berbahan karet* (We explained that a natural skin condom is a condom made of sheepskin/lambskin.)
13	A person will not get HIV if she or he is taking antibiotics	*Seseorang tidak akan terkena HIV jika dia menggunakan antibiotik*	*Seseorang tidak akan tertular HIV selama dia menggunakan antibiotik. Contoh antibiotik: ampisilin, amoksisilin, dan sebagainya* (We included examples of the most commonly used antibiotics, such as ampicillin and amoxicillin.)

### Item analysis

Item number 14 was answered correctly by nearly all the participants (97.1%) (Table 3). On the other hand, based on our analysis, item number 12 was the most difficult item with less than 30% of participants correctly answering this item. This finding was in line with our Atlas.ti review that the fewest participants reported item number 14 as the most difficult statement to answer while the items about condoms (including item number 12) were the most difficult, according to participants. The other 16 items were acceptable since the percentage of correct answers was between 30 and 80%. Four items (no 4, 11, 14, and 17) had corrected item-total correlation lower than 0.3, and only two items (no 11 and 17) were lower than 0.25.

**Table 3:** Item analysis of HIV-KQ-18 Bahasa Indonesia

Item		Percentage of correct answers	Corrected item-total correlation
1	Coughing and sneezing do not spread HIV	70.1	0.340
2	A person can get HIV by sharing a glass of water with someone who has HIV	69.6	0.441
3	Pulling out the penis before a man climaxes/cums keeps a woman from getting HIV during sex	63.4	0.403
4	A woman can get HIV if she has anal sex with a man	75.5	0.270
5	Showering, or washing one's genitals/private parts, after sex keeps a person from getting HIV	57.8	0.476
6	All pregnant woman infected with HIV quickly show serious signs of being infected	35.9	0.496
7	People who have been infected with HIV quickly show serious signs of being infected	56.6	0.483
8	There is a vaccine that can stop adults from getting HIV	63.4	0.497
9	People are likely to get HIV by deep kissing, putting their tongue in their partner’s mouth, if their partner has HIV	44.1	0.405
10	A woman cannot get HIV if she has sex during her period	76.5	0.493
11	There is a female condom that can help decrease a woman's chance of getting HIV	56.7	0.226
12	A natural skin condom works better against HIV that does a latex condom	24.8	0.382
13	A person will not get HIV if she or he is taking antibiotics	72.3	0.546
14	Having sex with more than one partner can increase a person's chance of being infected with HIV	97.1	0.253
15	Taking a test for HIV one week after having sex will tell a person if she or he has HIV	34.3	0.427
16	A person can get HIV by sitting in a hot tub or a swimming pool with a person who has HIV	73.9	0.497
17	A person can get HIV from oral sex	67.5	0.175
18	Using vaseline or baby oil with condoms lowers the chance of getting HIV	49.5	0.416

### Validation

Based on known-group validity, the HIV knowledge of participants with bachelor (11.0 ± 3.9) and postgraduate (13.1 ± 3.6), educational background in health sciences (12.0 ± 3.6), experience in attending a workshop about HIV (11.9 ± 3.8) and higher monthly expense > 5 million (12.2 ± 3.9) had significant higher HIV knowledge than participants with education level up to senior high school (9.6 ± 4.2), without educational background in health sciences (9.7 ± 4.2), never experienced attending a workshop about HIV (10.3 ± 4.0) and monthly expense (2 million (10.5 ± 4.0) (Table 4).

**Table 4:** Known-group validity of HIV-KQ-18

Variable	HIV-KQ-18 score		*p-*value
	Mean	SD	
Education level			
Up to senior high school	9.6	4.2	< 0.001^a^
Bachelor	11.0	3.9	
Postgraduate	13.1	3.6	
Having educational background in health sciences			
No	9.7	4.2	< 0.001
Yes	12.0	3.6	
Have attended workshop(s) about HIV			
No	10.3	4.0	< 0.001
Yes	11.9	3.8	
Monthly expense (in Rupiah)			
< 2 million	10.5	4.0	< 0.001^b^
2–3 million	11.3	3.7	
3–4 million	11.6	4.0	
4–5 million	11.7	4.1	
> 5 million	12.2	3.9	

^a^A post hoc analysis showed that the difference between these three educational levels were significant (*p* < 0.001)

^b^The difference was only significant between participants with monthly expenses > 5 million Rupiah and < 2 million Rupiah

**Fig. 2:**
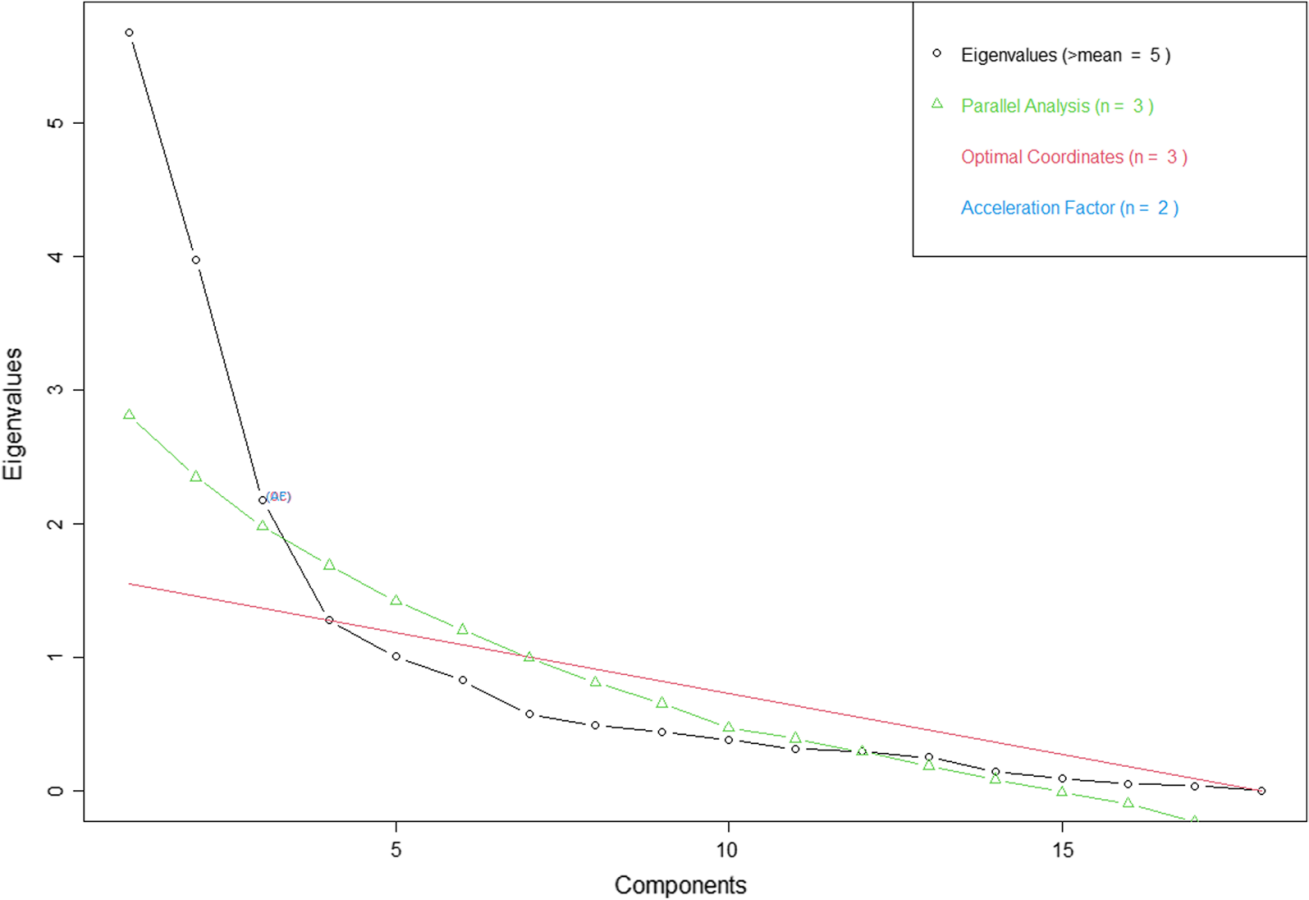
The non-graphical solution of Scree plot to determine the number of factors to be retained

In the EFA, based on eigenvalues, 5 factors could be retained, only 3 factors based on parallel analysis and optimal coordinates, while 2 factors based on acceleration factor (Fig. 2). However, the interpretation of 3 or 5 factors was very difficult and some items were cross-loading. Furthermore, in the model with 5 factors, one factor only had 1 item. Therefore, two factors, with each contained of 9 items, were retained from the EFA with RMSEA value of 0.133 and RMSR value of 0.06. No item had loading factors lower than 0.30. Three items (no 8, 9, 17) had a loading factor higher than 0.30 in both factors. The details of the EFA are presented in Table 5.

**Table 5:** Factor analysis of HIV-KQ-18 Bahasa Indonesia

Item	Description	Loading factor		Cronbach’s alpha
		Factor 1	Factor 2	
9	People are likely to get HIV by deep kissing, putting their tongue in their partner’s mouth, if their partner has HIV	0.97	0.33	0.75
2	A person can get HIV by sharing a glass of water with someone who has HIV	0.96	0.27	
16	A person can get HIV by sitting in a hot tub or a swimming pool with a person who has HIV	0.80	0.01	
7	People who have been infected with HIV quickly show serious signs of being infected	0.62	0.11	
6	All pregnant woman infected with HIV quickly show serious signs of being infected	0.54	0.25	
1	Coughing and sneezing do not spread HIV	0.49	0.06	
8	There is a vaccine that can stop adults from getting HIV	0.40	0.37	
15	Taking a test for HIV one week after having sex will tell a person if she or he has HIV	0.39	0.28	
11	There is a female condom that can help decrease a woman's chance of getting HIV	0.30	0.04	
17	A person can get HIV from oral sex	0.39	0.73	0.71
10	A woman cannot get HIV if she has sex during her period	0.18	0.66	
4	A woman can get HIV if she has anal sex with a man	0.15	0.62	
3	Pulling out the penis before a man climaxes/cums keeps a woman from getting HIV during sex	0.05	0.59	
18	Using vaseline or baby oil with condoms lowers the chance of getting HIV	0.08	0.58	
13	A person will not get HIV if she or he is taking antibiotics	0.30	0.57	
5	Showering, or washing one's genitals/private parts, after sex keeps a person from getting HIV	0.25	0.48	
14	Having sex with more than one partner can increase a person's chance of being infected with HIV	0.27	0.46	
12	A natural skin condom works better against HIV that does a latex condom	0.23	0.41	

The Cronbach’s alpha value of the first factor was 0.75, while the second factor was 0.70. These indicated these two factors had good reliability.

### Participants' statements about the most difficult item to answer

**Fig. 3:**
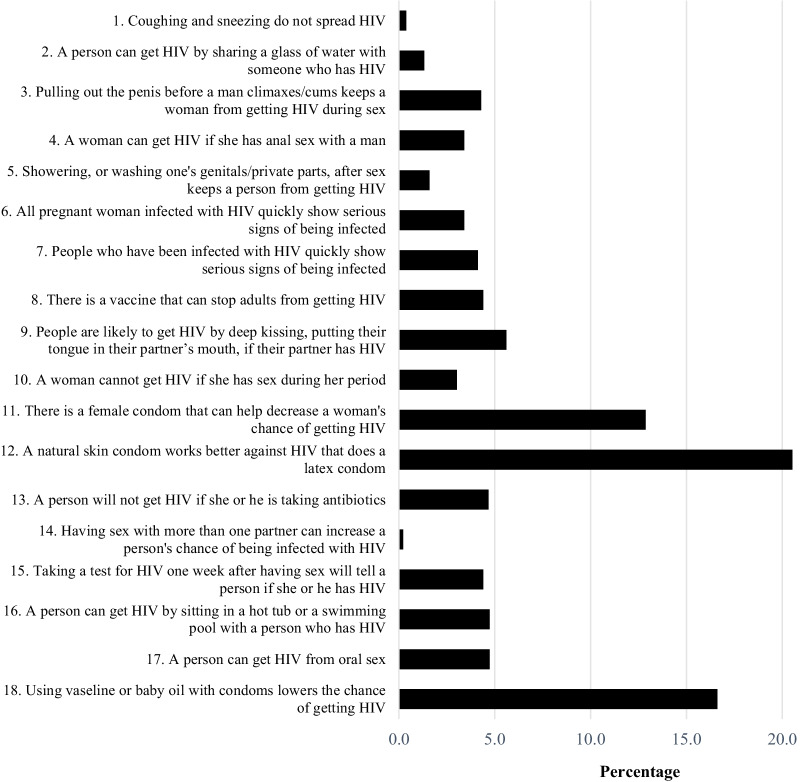
Questions which were the most difficult for participants to answer according to Atlas.Ti analysis

Of the 1,249 participants, 915 participants (73% response rate) responded to the question regarding which items were the most difficult to answer. It was important to highlight that most of the participants mentioned more than 1 item. Nearly half of the participants (45%) said the questions regarding condoms (items 11, 12 and 18) were the most difficult for them to answer (Fig. 3). Furthermore, 11% of those who answered said that the questions about the HIV/AIDS incubation period (items no 6, 7, and 15) were also difficult to answer. The most common reason given was a lack of HIV/AIDS education. Other findings in the study were 149 participants (16.3% from 915) stated that the instrument was very useful for determining a person's level of HIV/AIDS awareness because (i) to answer correctly, this instrument had to be read properly; and (ii) the sentence structure for each item was simple, clear and easy to understand.

## Discussion

Our findings suggest that the HIV-KQ-18 Bahasa Indonesia is a reliable and valid instrument for use in a general Indonesian population. The instrument's adaptation phase indicates that adding a few words or examples to explain the context of each item is extremely beneficial to participants' understanding. This approach has also been applied in other adaptation and validation studies in Indonesia [33, 34]. Furthermore, based on factor analysis, we preferred to use two factors based on visual inspection of the scree tree.

This study is the first HIV-KQ-18 Bahasa Indonesia psychometric test study in Indonesia. Our results show that our adaptation test has expanded the use of the instrument in a broader general population compared to previous studies that used the instrument only for nurses [11] or people living with HIV [27]. In addition, participants were given an opportunity to report which statements were the most difficult for them. The majority of participants reported difficulty in responding to items regarding condoms, while others confessed to knowing little about HIV/AIDS. Other than sexual intercourse, some participants have ambiguity on how HIV/AIDS is transmitted and how to reduce the risk of getting HIV/AIDS. In the study, 97% of participants correctly answered item number 14, indicating that the majority of participants believed that HIV/AIDS is closely linked to sexual activity with more than one partner. However, more than half of the participants still believe that deep kissing with a partner with HIV did not transmit HIV, and using Vaseline or baby oil can be used with condoms to lower the risk of getting HIV.

HIV-KQ-18 Bahasa Indonesia can differentiate the HIV knowledge levels of participants according to education level, educational background, having experience attending a workshop about HIV and monthly expenses. Participants with higher education level, educational background in health sciences, experience in attending a workshop about HIV and higher monthly expense significantly had higher HIV knowledge than participants with lower education level, without educational background in health sciences, never experience in attending a workshop about HIV and lower monthly expense. These findings support the a priori hypothesis and known-group validity.

For construct validity, different considerations lead to different number of factors to be retained. We decided to retain 2 factors although the original version HIV-KQ-45 suggested only 1 factor [13]. The first factor tends to focus on the HIV transmission outside sexual intercourse, excepts 2 items, namely “taking a test for HIV one week after having sex will tell a person if she or he has HIV” and “there is a female condom that can help decrease a woman's chance of getting HIV”. On the other hand, the second factor describes about HIV transmission due to sexual intercourse, except 1 item “a person will not get HIV if she or he is taking antibiotics. The RMSEA value is indicated that the model has low goodness of fit but the RMSR value is closed to 0.05 indicated the model has acceptable validity. We assume that the structural validity of the HIV-KQ18 is acceptable, although further investigation is necessary. The corrected item-total correlation shows that questions number 4, 11, 14, 17 had a corrected item-total correlation lower than 0.3. The questions are related to the type of sexual intercourse, the use of female condom and change of sex partner which may be uncommon in our country. The culture, law and religion in Indonesia, also support the normal sexual intercourse with single partner. Based on this situation, we recommend to supervise the participant during the application of this instrument in the future research.

Three items had cross loading factors between 2 factors. Since the development of HIV-KQ-18 also mandates that the instrument should also include items regarding sexual risk behaviours [13]; therefore, we suggest that these items need more explanations. For example, item no 17 needs more information about the meaning of “oral sex”. If this instrument is used as self-assessment instrument, a synonym of oral sex should be added to this item. If this instrument used as a part of interview, the interviewer should add an explanation about the meaning of oral sex.

Our study has both strengths and limitations. The strengths of the study are that it is the first adaptation and validation study of HIV-KQ-18 with over 1,000 participants from all main islands in Indonesia, and data collection was obtained from six main regions in Indonesia. The study provides evidence of validity and reliability in the range of participants' educational backgrounds from low to high educational background. The limitation of the study is that since the instrument was distributed through an online link, there is a potential participant bias that people with lower education and poor literacy levels would not participate in it. This is indicated by most participants are people with a bachelor degree, while only 25.2% of participants have an educational background of up to senior high school. Since people with poor literacy levels may have lower knowledge about HIV/AIDS, our findings may be slightly higher than the real public knowledge of HIV/AIDS. However, the study covered the two age groups with the largest number of people infected with HIV in the last ten years, i.e. 25–49 years (roughly 70%) and 20–24 years (roughly 15%) [35]. Further studies are proposed to use the instrument in paper form to target participants with low literacy levels, especially those who are not connected to the internet or have low educational levels.

## Conclusions

Based on psychometric analysis, HIV-KQ-18 Bahasa Indonesia is a valid and reliable instrument to assess the level of HIV/AIDS knowledge in the Indonesian population. We encourage the use of this standard instrument in future research and its use as a reference for measuring HIV knowledge.

## Data Availability

The first and the corresponding author can be contacted for data for research purposes.

## Code availability

All data analysis software used in the study is licensed from the University of Groningen in the Netherlands.
